# Evidence of Differential HLA Class I-Mediated Viral Evolution in Functional and Accessory/Regulatory Genes of HIV-1

**DOI:** 10.1371/journal.ppat.0030094

**Published:** 2007-07-06

**Authors:** Zabrina L Brumme, Chanson J Brumme, David Heckerman, Bette T Korber, Marcus Daniels, Jonathan Carlson, Carl Kadie, Tanmoy Bhattacharya, Celia Chui, James Szinger, Theresa Mo, Robert S Hogg, Julio S. G Montaner, Nicole Frahm, Christian Brander, Bruce D Walker, P. Richard Harrigan

**Affiliations:** 1 Partners AIDS Research Center, Massachusetts General Hospital, Harvard Medical School, Boston, Massachusetts, United States of America; 2 Howard Hughes Medical Institute, Chevy Chase, Maryland, United States of America; 3 British Columbia Centre for Excellence in HIV/AIDS, Vancouver, British Columbia, Canada; 4 Microsoft Research, Redmond, Washington, United States of America; 5 Los Alamos National Laboratory, Los Alamos, New Mexico, United States of America; 6 Santa Fe Institute, Santa Fe, New Mexico, United States of America; 7 Department of Computer Science and Engineering, University of Washington, Seattle, Washington, United States of America; 8 Faculty of Health Sciences, Simon Fraser University, Burnaby, British Columbia, Canada; 9 Department of Medicine, University of British Columbia, Vancouver, British Columbia, Canada; National Institute of Allergy and Infectious Diseases, United States of America

## Abstract

Despite the formidable mutational capacity and sequence diversity of HIV-1, evidence suggests that viral evolution in response to specific selective pressures follows generally predictable mutational pathways. Population-based analyses of clinically derived HIV sequences may be used to identify immune escape mutations in viral genes; however, prior attempts to identify such mutations have been complicated by the inability to discriminate active immune selection from virus founder effects. Furthermore, the association between mutations arising under in vivo immune selection and disease progression for highly variable pathogens such as HIV-1 remains incompletely understood. We applied a viral lineage-corrected analytical method to investigate HLA class I-associated sequence imprinting in HIV protease, reverse transcriptase (RT), Vpr, and Nef in a large cohort of chronically infected, antiretrovirally naïve individuals. A total of 478 unique HLA-associated polymorphisms were observed and organized into a series of “escape maps,” which identify known and putative cytotoxic T lymphocyte (CTL) epitopes under selection pressure in vivo. Our data indicate that pathways to immune escape are predictable based on host HLA class I profile, and that epitope anchor residues are not the preferred sites of CTL escape. Results reveal differential contributions of immune imprinting to viral gene diversity, with *Nef* exhibiting far greater evidence for HLA class I-mediated selection compared to other genes. Moreover, these data reveal a significant, dose-dependent inverse correlation between HLA-associated polymorphisms and HIV disease stage as estimated by CD4^+^ T cell count. Identification of specific sites and patterns of HLA-associated polymorphisms across HIV protease, RT, Vpr, and Nef illuminates regions of the genes encoding these products under active immune selection pressure in vivo*.* The high density of HLA-associated polymorphisms in *Nef* compared to other genes investigated indicates differential HLA class I-driven evolution in different viral genes. The relationship between HLA class I-associated polymorphisms and lower CD4^+^ cell count suggests that immune escape correlates with disease status, supporting an essential role of maintenance of effective CTL responses in immune control of HIV-1. The design of preventative and therapeutic CTL-based vaccine approaches could incorporate information on predictable escape pathways.

## Author Summary

One of the greatest challenges facing HIV-1 vaccine design today is the formidable capacity of the virus for mutation and adaptation, a characteristic that has contributed to the extensive worldwide genetic variability of HIV-1 strains observed today. On an individual basis, evolutionary selective pressures imposed by each infected person's unique immune response results in the selection and outgrowth of viral “escape” mutants capable of evading immune recognition, while on a population basis, complex evolutionary selective pressures imposed by the highly polymorphic genes of the human immune system shape HIV-1 diversity on a global level. Making sense of the seemingly infinite complexity of HIV immune escape is of paramount importance in our goal of developing a successful HIV vaccine. The current study uses cutting-edge statistical methods to identify specific sites and patterns of human leukocyte antigen (HLA) class I-restricted escape mutations in various HIV genes. Researchers summarize their findings in the form of “immune escape maps,” which highlight the differential contribution of immune imprinting to HIV genetic diversity, as well as identify specific sites in the viral genome under active immune selection pressure. Results from the present study contribute to our understanding of how human immune selective pressure contributes to variation in different HIV genes, and could help inform the development of HIV vaccines that take into consideration viral diversity.

## Introduction

Genetic variation within the highly polymorphic human leukocyte antigen (HLA) class I region contributes to diversity of pathogen recognition by cytotoxic T lymphocytes (CTLs) [1], and acts as a selective force shaping viral evolution within an infected host [2–6] through selection of mutations that allow the virus to escape recognition by HLA-restricted CTLs [5,7–9]. Immune escape may also represent a significant force shaping viral evolution at the population level through an HLA “imprinting effect,” in which escape mutations selected in the context of common HLA class I alleles may become predominant in the circulating viral population if they do not revert upon transmission to new hosts [2,10,11].

One of the major challenges to HIV vaccine design is the extensive worldwide sequence diversity of this pathogen, fueled in part by the extreme mutational capacity of the virus [12]. However, despite this considerable diversity, evidence indicates that there are constraints on viral evolution [2,13,14], and that escape in response to specific immune selective pressures (similar to escape from drug selective pressures [15]) follows broadly predictable mutational patterns [13,14]. A comprehensive identification of specific sites and patterns of immune escape in clinical HIV-1 isolates will further our understanding of how immune selection contributes to viral diversity [2,16] and will also identify specific viral regions under active immune selection pressure, thus providing information relevant to the selection of candidate immunogens for an HIV-1 vaccine.

Improvements in DNA sequencing technologies and the availability of large cohorts of HIV-1 infected individuals now allow us to employ population-based genetic association approaches to identify viral amino acids (aa) under active immune selection pressure in vivo [2]; however, methodological challenges associated with identifying such mutations are now recognized [16]. Moore et al. [2] were the first to identify HLA-class I-associated polymorphisms across codons 20–227 of HIV-1 reverse transcriptase (RT) in a large clinically derived dataset using a Chi-squared association approach, thus providing evidence for HLA class I-mediated viral evolution on a population level. However, the application of standard statistical tests is inappropriate for the analysis of viral isolates with a shared phylogenetic history, since descent from a common ancestor means that viral sequences may not be treated as statistically independent entities [17]. Specifically, a cause for concern is the application of standard statistical methods to identify HLA-associated viral polymorphisms in cohorts comprising individuals of diverse genetic backgrounds (sampled from populations with differential HLA allele distributions) infected with heterogeneous viral strains. In this case, standard statistical approaches such as the Chi-squared test may identify confounding associations between strain- or lineage-specific viral polymorphisms and specific HLA alleles that are over-represented in subpopulations of individuals harboring infections with those strains. In this case, the observed “HLA-associated polymorphism” is not evidence of active HLA-mediated immune selection. Rather, the association is simply a statistical correlation between possession of a particular HLA allele observed among persons of a particular ethnic background, and a lineage-specific viral polymorphism, arising as a result of descent from a common ancestor (“founder effect”) [16]. The use of population-based, viral lineage-corrected analyses, such as those recently developed by Bhattacharya et al. [16], are therefore essential in order to accurately identify sites of active immune selection in the genomes of sequence-diverse pathogens such as HIV-1.

In addition, although there is clear evidence supporting HIV-1 adaptation to HLA class I-mediated CTL selection pressure from an evolutionary standpoint [2–5], the relevance of immune escape to clinical HIV disease progression remains unclear, due in part to the fact that many studies have focused small numbers of participants and/or escape within a limited number of HLA-restricted CTL epitopes in the viral genome [3–6,18–20]. Furthermore, no studies to date have linked HIV disease progression to HLA-associated polymorphisms corrected for lineage effects. Here we identify lineage-corrected [16] HLA class I-associated polymorphisms across select functional and accessory/regulatory HIV-1 genes in a cross-sectional analysis of a large cohort of chronically infected, treatment-naïve individuals, and investigate the relationship between these polymorphisms and clinical markers of HIV disease.

## Results

**Figure 1:**
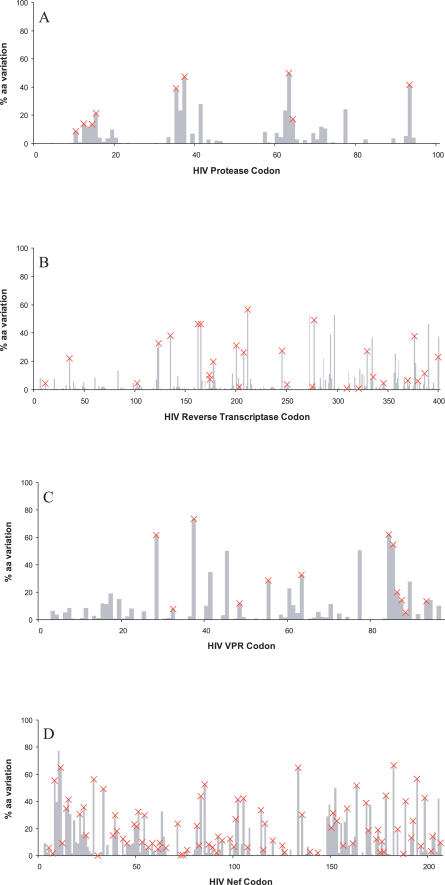
Differential Density of HLA Class I-Associated Viral Polymorphisms across HIV Protease, Reverse Transcriptase, Vpr, and Nef Polymorphism maps display the percentage amino acid variation over protease (A), reverse transcriptase (B), Vpr (C), and Nef (D). Red × symbols indicate codons at which at least one significant HLA-associated polymorphism was observed with *q* < 0.2.

A large, well-characterized cohort of chronically HIV-1 infected, antiretroviral drug-naïve individuals from British Columbia, Canada [21], for whom HLA class I typing and HIV RNA genotyping of select functional and accessory/regulatory genes were performed, was used to identify HLA class I allele-associated viral polymorphisms across a 499 aa fragment spanning protease and most of RT p51 (*n* = 532 successfully genotyped), 96 aa of Vpr (*n* = 425), and 206 aa of Nef (*n* = 686). HLA class I allele-associated viral polymorphisms were identified using analytical approaches described in [16], which feature a correction for viral lineage effects by adjusting for phylogenetic relationships between sequences [16], and a correction for multiple comparisons using a *q*-value approach [22], which sets the false-discovery rate (20% with *q* < 0.2) among significant associations. The level of variation at single residues in protease, RT, Vpr, and Nef ranged from 0% to maxima of 50%, 57%, 73%, and 77% respectively (Figure 1), while the mean pairwise amino acid identity for these same genes (calculated as the percentage of codons exhibiting identical amino acids for each pairwise combination of sequences) was 92.9%, 95.3%, 89.0%, and 83.1%, respectively, indicating typical intrasubtype levels of HIV sequence diversity in this cohort of relatively homogeneous subtype distribution (97.5% HIV-1 subtype B).

It is important to note that the phylogenetically corrected methods for identification of HLA-associated viral polymorphisms developed by Bhattacharya et al. [16] do more than simply correct for confounding due to HIV intersubtype (or interclade) variation. Even among clade-homogeneous datasets, “subclade” lineage-specific effects may yield confounding associations with HLA alleles, especially if the cohort is composed of subpopulations with differential HLA allele distributions. Indeed, there was clear evidence of phylogenetic subclusters within subtype B sequences in this cohort (Figure S1).

We therefore compared phylogenetically corrected methods to a simple uncorrected test (simple Fisher), and found that even in this predominantly subtype B-infected cohort, HLA-associated polymorphisms identified using phylogenetically corrected methods had higher fractions of associations that could be independently validated by immunological data than those defined using a simple Fisher exact test (unpublished data). An example of a case in which an apparent HLA-associated polymorphism identified using a simple Fisher exact test represents an artifact of the phylogenetic tree is illustrated in Figure S1. In this analysis, therefore, we report only HLA-associated polymorphisms defined using phylogeny-based methods [16].

Application of the viral lineage-corrected method [16] yielded a total of 478 unique HLA allele-associated viral polymorphisms with *q* < 0.2 across the genes investigated (Table S1). These occurred at nine (9%), 28 (7%), 12 (12.5%), and 84 (41%) unique codons in protease, RT, Vpr, and Nef, respectively, highlighting a dramatic variation in HLA-associated imprinting across HIV-1 genes (Figure 1). HLA-B alleles accounted for half (241 of 478; 50.4%) of the total number of HLA-associated polymorphisms, while HLA-A and C alleles accounted for 112 (23.4%) and 125 (26.2%), respectively.

Previous studies have validated the application of such genetic association analyses of large clinically derived datasets in order to identify HLA-restricted CTL escape mutations selected in vivo [16]. Knowing that with *q* < 0.2, about 20% of identified HLA-associated polymorphisms will represent false-positive results, we set about classifying the 478 identified polymorphisms into putative true-positive or false-positive results based on the strength of independent biological evidence supporting each polymorphism as an escape-associated mutation. The highest level of biological support was assigned to HLA-associated polymorphisms falling within or proximal to (± 3 aa) a published CTL epitope [23] restricted by that particular HLA allele, thereby supporting these associations as in vivo-selected mutations directly or indirectly affecting MHC binding, T cell receptor recognition and/or intracellular peptide processing [24–26]. A second level of support was assigned to those associations falling within or similarly proximal to putative/novel HLA-restricted epitopes, identified by scanning the cohort consensus sequence for HLA-restricted epitope anchor residue motifs using two independent bioinformatic tools (MotifScan [Los Alamos National Laboratory], http://www.hiv.lanl.gov/content/immunology/motif_scan/motif_scan; and Epipred [Microsoft Research], http://atom.research.microsoft.com/bio/epipred.aspx).

To provide further biological support for these associations we drew upon an independent cohort of 372 HIV-1 infected individuals screened for in vitro HLA-restricted, CTL-mediated interferon-gamma (IFN-γ) responses against a set of overlapping HIV-1 subtype B consensus peptides spanning the entire viral proteome using the IFN-γ enzyme-linked immunosorbent spot assay (ELISpot) [27], in order to identify HLA class I alleles significantly associated with CTL-mediated IFN-γ production in response to stimulation with consensus HIV peptides (see Methods). HLA allele-associated polymorphisms mapping within a significantly reactive HLA allele/HIV consensus peptide pair were identified as potential escape-associated mutations to known or novel HLA-restricted CTL epitopes.

**Figure 2:**
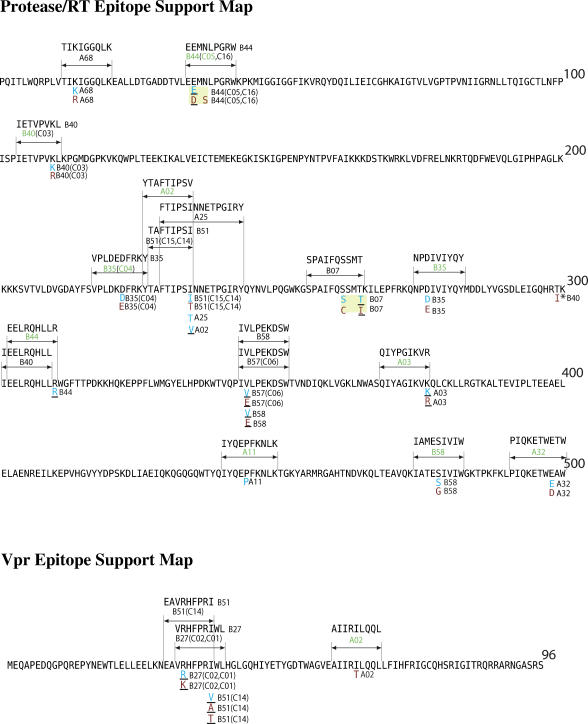
Epitope Support Maps: HLA-Associated Polymorphisms Mapping within or Directly Proximal to Published HLA-Restricted CTL Epitopes in Protease/Reverse Transcriptase and Vpr HLA-associated polymorphisms occurring within or directly proximal (± 3 aa) to published HLA-restricted CTL epitopes in protease/RT and Vpr are shown. Polymorphisms associated with the same HLA allele(s) occurring within, or directly proximal to (± 3 aa) published HLA-restricted epitope boundaries are boxed together in yellow. The published epitope sequence (and associated HLA) is indicated above the epitope boundary arrow. If associations within the same epitope boundaries are ascribed to several linked HLA alleles, the HLA that is known to present the epitope is indicated next to it. Red (“escape”) amino acids indicate positive correlations (amino acids that are enriched in the presence of a specific HLA allele). Thus, “escape” (red) amino acids presumably reflect the escape variant specific for that HLA allele. Blue (“reversion”) amino acids indicate negative correlations, where the presence of a specific HLA allele is associated with the *absence* of a particular amino acid at a specific position—or, likewise, where the absence of the specific HLA allele is associated with the presence of this amino acid. Thus, “reversion” (blue) amino acids presumably reflect the immunologically susceptible form specific for that HLA allele, and the amino acid to which the sequence may revert upon transmission to an HLA-unmatched host. Underlined associations indicate potential HLA anchor residue sites. Proximal associations (within ± 3 aa of epitope boundaries) are marked with an asterisk (*). Associations additionally supported by functional IFN-γ (ELISpot) data are indicated in green.

**Figure 3:**
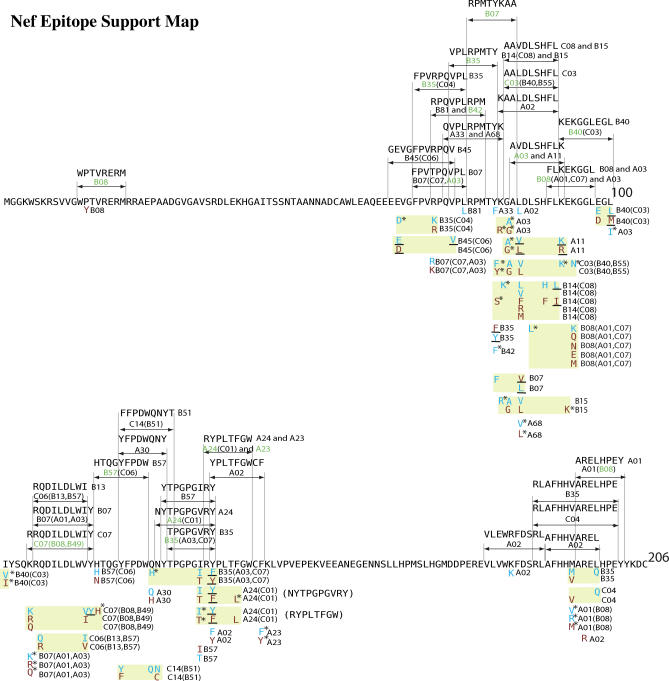
Epitope Support Map: HLA-Associated Polymorphisms Mapping within or Directly Proximal to Published HLA-Restricted CTL Epitopes in Nef HLA-associated polymorphisms occurring within or directly proximal (± 3 aa) to published HLA-restricted CTL epitopes in Nef are shown. Polymorphisms associated with the same HLA allele(s) occurring within, or directly proximal to (± 3 aa) published HLA-restricted epitope boundaries are boxed together in yellow. The published epitope sequence (and associated HLA) is indicated above the epitope boundary arrow. If associations within the same epitope boundaries are ascribed to several linked HLA alleles, the HLA that is known to present the epitope is indicated next to it. Red (“escape”) amino acids indicate positive correlations (amino acids that are enriched in the presence of a specific HLA allele). Thus, “escape” (red) amino acids presumably reflect the escape variant specific for that HLA allele. Blue (“reversion”) amino acids indicate negative correlations, where the presence of a specific HLA allele is associated with the *absence* of a particular amino acid at a specific position—or, likewise, where the absence of the specific HLA allele is associated with the presence of this amino acid. Thus, “reversion” (blue) amino acids presumably reflect the immunologically susceptible form specific for that HLA allele, and the amino acid to which the sequence may revert upon transmission to an individual lacking that HLA allele. Underlined associations indicate potential HLA anchor residue sites. Proximal associations (within ± 3 aa of epitope boundaries) are marked with an asterisk (*). Associations additionally supported by functional IFN-γ (ELISpot) data are indicated in green

**Figure 4:**
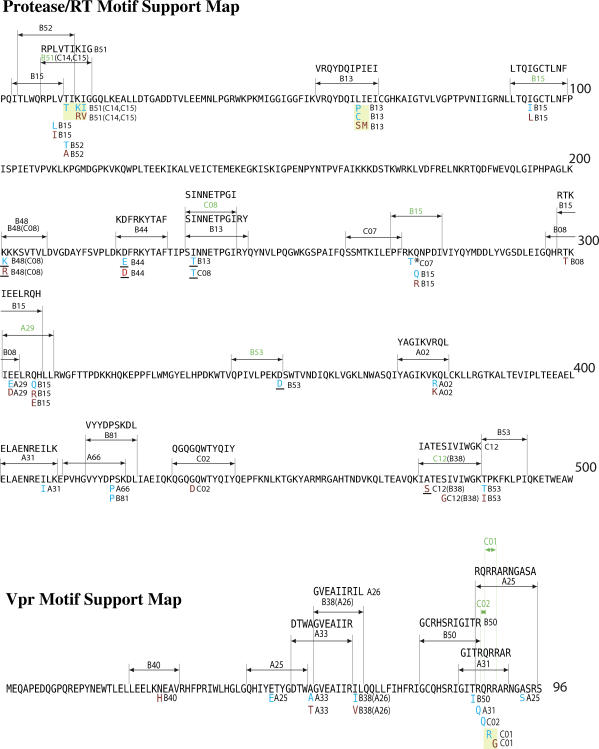
Motif Support Maps: HLA-Associated Polymorphisms Mapping within or Directly Proximal to HLA-Restricted CTL Epitope Motifs in Protease/Reverse Transcriptase and Vpr HLA-associated polymorphisms occurring within or directly proximal to (± 3 aa) known HLA-restricted epitope anchor residue motifs in protease/RT and Vpr are shown. Polymorphisms associated with the same HLA allele(s) occurring within or directly proximal to (± 3 aa) of HLA-restricted epitope anchor residue motifs are boxed together in yellow. Predicted epitopes (where available) are indicated above the epitope boundary arrow, while HLA-restricted epitope motif boundaries are simply marked by their corresponding HLA association. Where multiple overlapping motifs were present, only the most commonly observed one (based on analysis of individual sequences) is indicated. Linked alleles are indicated in brackets, and in ranked order based on p-value of the association. Red (“escape”) amino acids indicate positive correlations (amino acids that are enriched in the presence of a specific HLA allele thus presumably represent escape variants). Blue (“reversion”) amino acids indicate negative correlations, where the presence of a specific HLA allele is associated with the *absence* of a particular amino acid at a specific position—and vice versa—in this case, the amino acid presumably reflects the immunologically susceptible form specific for that HLA allele. Underlined associations indicate potential HLA anchor residue sites. Proximal associations (within ± 3 aa of epitope boundaries) are marked with an asterisk (*). Associations supported by functional IFN-γ (ELISpot) data are indicated in green. Where an association is supported solely by ELISpot data, but no predicted epitope or motif, the arrow and lines are all green.

**Figure 5:**
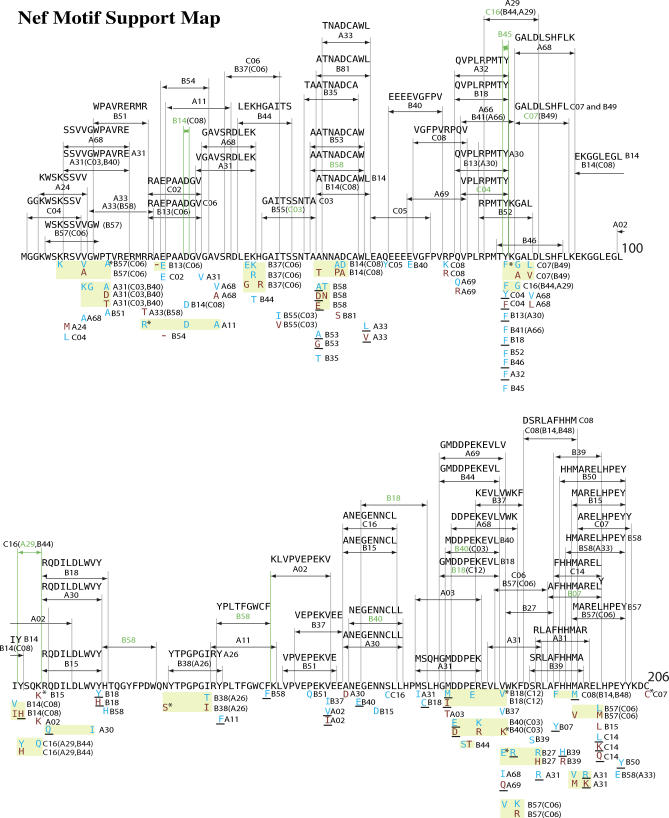
Motif Support Maps: HLA-Associated Polymorphisms Mapping within or Directly Proximal to HLA-Restricted CTL Epitope Motifs in Nef HLA-associated polymorphisms occurring within or directly proximal to (± 3 aa) known HLA-restricted epitope anchor residue motifs in Nef are shown. Polymorphisms associated with the same HLA allele(s) occurring within or directly proximal to (± 3 aa) of HLA-restricted epitope anchor residue motifs are boxed together in yellow. Predicted epitopes (where available) are indicated above the epitope boundary arrow, while HLA-restricted epitope motif boundaries are simply marked by their corresponding HLA association. Where multiple overlapping motifs were present, only the most commonly observed one (based on analysis of individual sequences) is indicated. Linked alleles are indicated in brackets, and in ranked order based on p-value of the association. Red (“escape”) amino acids indicate positive correlations (amino acids that are enriched in the presence of a specific HLA allele thus presumably represent escape variants). Red dashes indicate positions where the presence of the HLA is associated with deletion of the amino acid at that position. Blue (“reversion”) amino acids indicate negative correlations, where the presence of a specific HLA allele is associated with the *absence* of a particular amino acid at a specific position—and vice versa—in this case, the amino acid presumably reflects the immunologically susceptible form specific for that HLA allele. Underlined associations indicate potential HLA anchor residue sites. Proximal associations (within ± 3 aa of epitope boundaries) are marked with an asterisk (*). Associations supported by functional IFN-γ (ELISpot) data are indicated in green. Where an association is supported solely by ELISpot data, but no predicted epitope or motif, the arrow and lines are all green.

**Figure 6:**
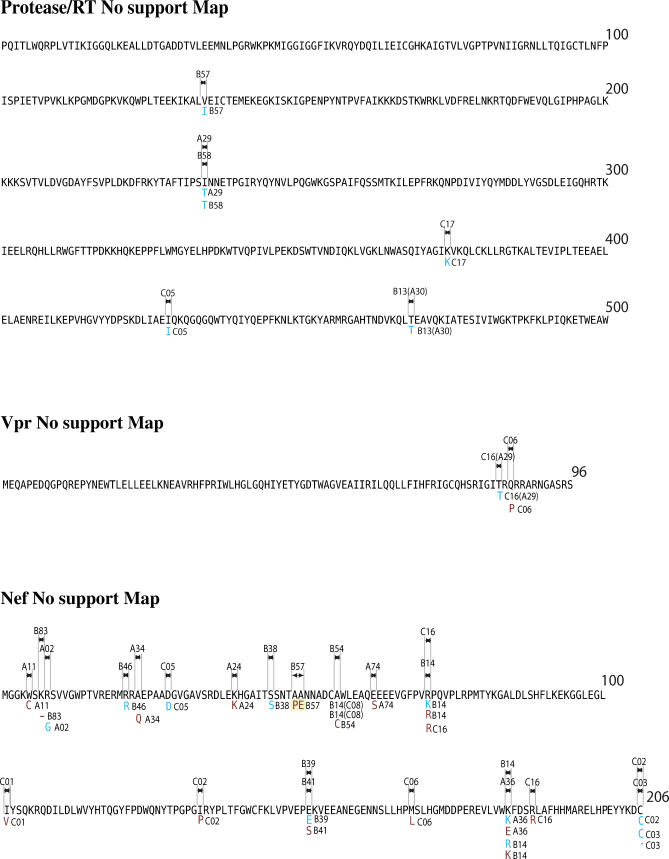
No Support Maps: HLA-Associated Polymorphisms in Reverse Transcriptase, Vpr, and Nef That Do Not Map within Known or Putative Epitopes, and Are Not Supported by Functional IFN-γ (ELISpot) Data Associations displayed on this map are likely to be highly enriched for the expected proportion of false-positives (20% with *q* < 0.2). However, we cannot rule out the possibility that these may represent processing escape mutations occurring distant from the epitope site, compensatory mutations, unusual epitopes, or other factors. Note that no “unsupported” HLA-associated polymorphisms were observed in HIV protease.

Finally, we grouped together HLA allele-specific associations clustering within these epitopes or motifs, and paired together alleles in linkage disequilibrium (Table S2) associated with the same HIV polymorphism(s), to create a series of immune escape maps capturing the minimum number of HLA-restricted epitopes and/or motifs required to explain the data (Figures 2–5). Associations that did not map within a known epitope or motif, and were not supported by ELISpot data or attributable to HLA allele linkage, were listed in a separate map (Figure 6).

After pairing together linked alleles, approximately 35% of codons in protease, RT, Vpr, and Nef exhibiting HLA-associated polymorphisms mapped inside (*n* = 77; 81%) or within ± 3 aa (*n* = 18; 19%) of a published CTL epitope specific to that HLA allele (Figures 2 and 3). Significant associations were collapsed into two categories based on the direction of the HLA selection pressure: amino acids enriched in the presence of a specific allele (positive or “escape” correlations, presumably representing the escape variant for that allele), and amino acids depleted in the presence of a specific allele (negative or “reversion” correlations, presumably representing the immunologically susceptible or “wild-type” form for that allele, and also representing the amino acid to which the residue will likely revert to upon transmission an individual lacking that allele). Overall, the majority of HLA-associated polymorphisms (58% of epitope-supported associations) represent negative (“reversion”) correlations (*p* = 0.002). Note that detection of a “reversion” correlation in the absence of an associated “escape” correlation may arise in the case where a specific allele selects for multiple amino acids at a given position, creating a situation where there may be sufficient statistical power to detect the “reversion” correlation but not to identify all possible escape variants.

A considerable number of codons exhibit multiple HLA associations, particularly in Nef. A total of 57 multiple associations were observed, with 2, 6, 3, and 46 occurring across protease, RT, Vpr, and Nef, respectively. In 23 of these 57 cases (for example, *Nef* codons 81 and 135), the same amino acid represents an escape variant for one HLA allele, but the susceptible form for another, highlighting a “tug-of-war” of differential HLA selective pressures contributing to populational HIV sequence diversity at specific codons.

There were dramatic differences in the number of HLA-associated polymorphisms across the genes investigated. Not only did Nef exhibit a much higher density of epitope-supported associations compared to protease/RT and Vpr, but the escape patterns also tended to be more complex in *Nef* than in other genes. A total of 53% of escaping epitopes in Nef exhibited HLA-associated polymorphisms at multiple positions within the epitope, compared with 12% and 0% in protease/RT and Vpr, respectively (*p* = 0.004). Similarly, epitope-proximal associations (occurring within 3 aa of a published epitope) were also observed more frequently in Nef (*n* = 16 [22%]) while occurring only relatively rarely in protease/RT/Vpr (total *n* = 2 [9%]), although this did not achieve statistical significance (*p* = 0.2).

Overall, HLA-associated polymorphisms were observed with relatively equal frequency across all positions within published HLA-restricted epitopes. There was no statistically significant enrichment for HLA-associated polymorphisms at anchor residues (generally defined as epitope residues 2 and C-terminal with some exceptions [28,29]) over other residues in Nef (*p* = 0.7) or protease/RT/Vpr (*p* > 0.1) suggesting that amino acid changes potentially affecting peptide binding to HLA class I molecules are not a favored mechanism of escape.

We organized a further ~50% of the identified associations into “motif-support” maps (Figures 4 and 5) that grouped HLA-associated polymorphisms within HLA-restricted epitope anchor residue motifs identified by scanning the cohort consensus sequence. Based on evidence that HLA-associated polymorphisms identified in genetic association studies predict the location of previously uncharacterized epitopes [16], we would expect that a substantial proportion of motif-supported associations represent escape mutations within novel epitopes, a hypothesis supported by the fact that many motif-supported associations (40%, 31%, 22%, and 19% in protease, RT, Vpr, and Nef, respectively) are substantiated by in vitro IFN-γ ELISpot responses to HIV-specific consensus peptides containing these motifs. Consistent with observations drawn from the epitope-support maps (Figures 2 and 3), the majority (63%) of associations in the motif-support maps represent “reversion” associations, with a much more complex pattern of escape observed in Nef compared to protease/RT/Vpr.

The remaining ~15% of HLA-associated polymorphisms did not map to known epitopes and were unlikely to lie within or proximal to novel epitopes as suggested by in vitro IFN-γ ELISpot responses or bioinformatic motif scans (Figure 6). Although these proportions are consistent with the false-discovery rate of ~20% (*q* < 0.2), lack of biological support cannot be used to definitively categorize these as “false-positive” associations in any particular case. In some cases, these may represent processing escape mutations occurring distant from the epitope site, compensatory mutations, unusual epitopes, or other factors. Similarly, HLA-associated polymorphisms mapping within an HLA-matched epitope or motif are likely highly enriched for mutations directly or indirectly conferring immune escape, but likely contain smaller numbers of false-positive associations as well.

**Figure 7:**
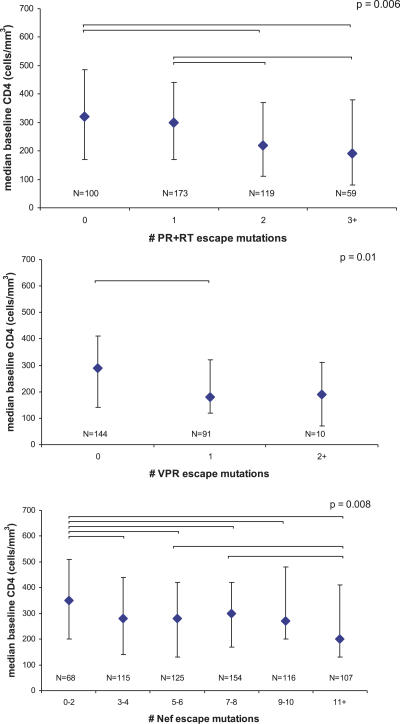
Inverse Dose-Response Relationship between Accumulation of Epitope-Associated Escape Mutations in Protease/Reverse Transcriptase, Vpr, and Nef and CD4^+^ Cell Count in Chronic Untreated HIV Infection A significant inverse dose-response relationship is observed between the presence of epitope-associated escape mutations in protease/RT (A), Vpr (B), and Nef (C) and CD4^+^ cell count in chronic untreated HIV infection. Escape mutations were defined as all significant HLA/HIV associations which mapped within or proximal to (± 3 aa) known CTL-restricted epitopes (listed in Figures 2 and 3). Analysis was restricted to participants able to exhibit “escape,” i.e., those possessing at least one HLA allele featured in Figures 2 and 3 (for each gene investigated). Linked alleles were removed to avoid double counting of associations. Diamonds and bars represent median and interquartile ranges, respectively, of pretherapy CD4^+^ cell count for each of the associated escape strata. Overall *p*-value obtained using the Kruskal-Wallis test. Significant (*p* < 0.05) pairwise associations are indicated by brackets.

Although there is clear evidence documenting the selection of escape variants over the course of HIV infection [3–5,7–9], the clinical significance of immune escape remains incompletely understood [18–20]. Moore et al. reported a significant association between HLA-associated polymorphisms and plasma viral load [2]; however, no studies to date have linked lineage-corrected HLA-associated polymorphisms with markers of disease progression on a population basis. We therefore investigated correlations between the presence of HLA-associated polymorphisms and clinical status in chronic untreated infection as measured by pretherapy CD4^+^ cell number and plasma viral load. In order to adopt the most conservative definition of “escape,” the primary analysis was restricted to those amino acid associations mapping inside or within ± 3 aa of a known HLA-restricted CTL epitope (Figures 2 and 3). A significant inverse dose–dependent relationship was observed between the median pretherapy CD4^+^ cell count and the number of epitope-associated polymorphisms observed in protease/RT (*p* = 0.006), Vpr (*p* = 0.01), and Nef (*p* = 0.008) (Figure 7). A trend was observed between accumulation of epitope-associated polymorphisms in protease/RT (but not other proteins) and higher pretherapy viral load (*p* = 0.06 [unpublished data]). The dose-dependent association between epitope-associated polymorphisms and lower CD4^+^ cell counts supports the ability of large genetic association studies to identify biologically relevant in vivo CTL escape-associated mutations, but more importantly, supports a clinically relevant link between immune escape and HIV disease progression.

Note that the observed association between HLA-associated polymorphisms and lower CD4^+^ cell count is specific to HLA-associated polymorphisms mapping within or near published epitopes, and not simply a general association between viral mutations and HIV clinical status. In a secondary analysis we investigated correlations between the presence of motif-associated (Figures 4 and 5) and unsupported (Figure 6) polymorphisms and clinical parameters. A nonsignificant trend (*p* = 0.07) was observed between accumulation of motif-associated polymorphisms in protease/RT (but not other proteins) and lower median CD4^+^ cell counts, while no significant association was observed between clinical parameters and the presence of biologically unsupported associations, consistent with a stepwise enrichment for false-positives among associations in these categories.

## Discussion

The present study represents to our knowledge the largest population-based investigation of HLA class I-mediated imprinting on HIV sequence to date, as well as the first characterization of HLA-associated polymorphisms in each of a functional, accessory and regulatory gene. Results identify viral polymorphisms selected in vivo in context of a wide array of class I alleles. The confirmation of the B*1501-associated polymorphism at protease codon 93 reported by Bhattacharya et al. [16] and several reported by Moore et al. [2] in RT suggest that immune escape patterns in HIV-1 subtype B are consistent across the globe. The confirmation of several functionally verified CTL escape mutations previously observed in clinically derived isolates (including escape at residues 2, 8, 2, and 5 of the HLA-B*57 restricted IW9-RT [13,30], B*51-restricted TI8-RT [31], A*24-restricted RF10-nef [32], and B*08-restricted FL8-nef [5] epitopes, respectively) confirm the utility of genetic association studies to identify escape variants commonly selected in vivo. Taken together, results provide proof of principle that population-based approaches could complement smaller functional studies by providing a whole-gene or whole-virus picture of immune escape.

Results of this large-scale, multigene analysis reveal dramatically different levels of HLA-associated polymorphisms across HIV proteins, with a previously unreported, extraordinary density and complexity of HLA-associated polymorphisms in Nef. Nef exhibits considerable sequence diversity and thus may exhibit higher levels of mutational plasticity in response to selective pressures compared to genes exhibiting structural (e.g., Gag) or functional (e.g., protease/RT) constraints; however it is important to note that protease (and to a lesser extent RT) exhibit extensive mutational capacity under antiretrovirally mediated selection pressure [15], suggesting that mutational constraints on functional genes are unlikely to fully account for the relative paucity of HLA-associated polymorphisms across these regions. Rather, results are consistent with the density of CTL epitopes across these regions, as well as the relative immunogenicity of these proteins over the course of infection [27,33]. Limited data from longitudinal studies suggest that CTL escape mutations in *Nef* are selected earlier in infection [33,34], and thus, in a population of chronically infected individuals, one may expect a large burden of *Nef* escape mutations to have already accumulated. Note that, in the current study, *Nef* sequences were available for a larger number of participants, thus potentially increasing power to detect significant associations.

These data are also relevant to CTL-based HIV vaccine design. First and foremost, the analysis of clinically derived datasets identifies viral epitopes under active immune selection pressure, thus identifying in vivo immunogenic viral targets. The fact that we observed such a large number of HLA-associated polymorphisms, including many instances of specific codons apparently under diametrically opposed HLA-selective pressures (an observation consistent with Iversen et al. [35]), provide some evidence against the complete disappearance of all active viral epitopes under the HLA “imprinting hypothesis” (which states that escape mutations selected in response to the most common HLA alleles may become fixed in the circulating viral population [2,10], thus resulting in a potential loss of CTL responses to these epitopes and rendering them inappropriate as candidate immunogens). Taken together with evidence supporting rapid reversion of escape mutations after transmission to a new host [36], and the fact that escape mutations in one individual may represent the susceptible form in another [16], the “HLA imprinting effect” is unlikely to result in the creation of an immunologically refractive circulating viral population by eliminating all active CTL epitopes in this population. Rather, selection pressures mediated by diverse HLA class I alleles in HIV-1 infected populations appear to be actively contributing to viral diversity thus preserving a substantial number of immunologically active epitopes in the circulating population. These active epitopes, most notably those which exhibit the “push-and-pull” of diametrically opposed HLA selection pressures, could perhaps be incorporated into a CTL-based HIV-1 vaccine strategy.

The locations of HLA-associated polymorphisms relative to known or predicted HLA-appropriate epitopes revealed no statistically significant enrichment for mutations at epitope anchor residues versus other positions. Theoretically, if the predominant mechanism of CTL escapes were abrogation of peptide-MHC binding through anchor residue mutation, a polyvalent vaccine approach may have little merit. However, these observations, combined with previous documentation of de novo T cell responses arising in response to escape variants [37], strongly support the utility of incorporating viral sequence variation into immunogen design.

Given the adaptable nature of the CTL response [37], combined with the fact that the majority of reports of CTL escape to date have focused on small numbers of individuals and/or a select few epitopes [3–6,18–20,30–32,35], it is not surprising that the clinical consequences of CTL escape remain incompletely understood. Some studies report an association between selection of escape variants and loss of viremia control [20,38] and disease progression [6,18]; however, this does not seem to equally apply to all CTL epitopes [35,39]. Here we observe a significant, dose-dependent inverse relationship between HLA-associated mutations within published epitopes in functional and accessory/regulatory genes and lower CD4^+^ cell counts in chronic untreated HIV infection, thus supporting a link between presence of escape mutations and HIV disease status. Although detection of escape mutations indeed preceded a loss of immune control in previous case reports [6,18], it is important to note that the cross-sectional nature of the current study precludes any inferences regarding cause and effect. Likely, a longer duration of infection (among those with lower CD4^+^ cell counts in this cohort) may have facilitated the accumulation of CTL escape variants, a hypothesis we were unable to investigate, because seroconversion dates were generally unknown. Other limitations of this analysis include the inherent limitations associated with the use of a single CD4^+^ cell measurement in a cross-sectional study design, the lack of longitudinal HIV sequence data, as well as the fact that the cohort represents a group of individuals referred for antiretroviral treatment, and thus may be biased toward more rapid progression to disease. Despite these limitations, our findings support those of Moore et al. [2] who reported that HLA-associated polymorphisms in RT predicted plasma viral load (CD4^+^ cell counts were not investigated). At first, results appear inconsistent with those of Iversen et al. [35] who reported higher viral loads in patients with efficient CTL selection; however, results may be reconciled by the fact that the previous study [35] investigated clinical correlates of escape to a single HLA-restricted epitope, whereas the current study evaluates HLA-associated polymorphisms across multiple genes. Ideally, however, the relationship between selection of HLA-associated escape mutations and HIV disease progression should be addressed in an unbiased, longitudinal cohort study of untreated HIV-1 seroconverters for whom infection dates, viral loads and CD4^+^ T cell setpoints, and rates of disease progression are known.

Although a systematic in vitro characterization of novel CTL epitopes was beyond the scope of this manuscript, the observation that a substantial number of motif-associated polymorphisms are supported by HLA-restricted, peptide-specific IFN-γ responses in an ELISpot assay suggest that they represent escape mutations within uncharacterized epitopes [16]. As the locations of published epitopes tend to be biased toward conserved regions (due to the historic use of consensus or reference strains to construct peptide libraries), the “motif maps” could complement traditional epitope mapping by identifying epitopes located in more variable regions.

After controlling for the potentially confounding effects of viral lineage [16], strong evidence for HLA class I-mediated selection is observed across functional and accessory/regulatory HIV-1 genes, with up to 40% of residues in some HIV proteins (Nef, for example) exhibiting evidence for HLA-restricted immune selection. Our results thus confirm an active and substantial contribution of human immunogenetic selection pressure on viral evolution [2] and underscore the importance of understanding how HLA class I diversity drives HIV diversity. The observed correlation between the presence of HLA-associated CTL escape mutations and lower CD4^+^ cell counts supports the hypothesis that maintenance of effective CTL responses plays an important role in immune control of HIV infection, although further research in additional cohorts is needed. The observation that epitope anchor residue mutation appears not to be the predominant mechanism of CTL escape supports the incorporation of HIV sequence diversity in the development of preventative and therapeutic CTL-based vaccine approaches.

## Methods

### Study participants: The British Columbia HOMER cohort.

In British Columbia (BC), antiretroviral drugs are distributed free of charge to HIV-infected individuals through a centralized drug treatment program (for details, see [21]). The HAART Observational Medical Evaluation and Research (HOMER) cohort is an open cohort comprising all HIV-infected, antiretroviral-naïve adults who initiated HAART since August 1996 (*n* > 2,200 individuals enrolled to date). A subset of HOMER, comprising all treatment-naïve individuals who initiated HAART in BC between August 1996 and September 1999 (*n* = 1,191) has been described in detail previously [21]. Participants in the current cross-sectional study represent a nonrandom subset (*n* = 765; 64%) of these 1,191 individuals at baseline (prior to initiation of HAART) included based on the availability of a peripheral blood sample for HLA typing. A comparison of pre-therapy characteristics of those included (*n* = 765) and excluded (*n* = 426) reveals no significant differences in pretherapy CD4^+^ cell count (280 cells/mm^3^); however, those included had slightly lower pretherapy plasma viral load (pVL) (median 5.07 versus 5.15 log_10_ copies HIV RNA/ml, *p* = 0.03), were on average slightly older (median 37.2 versus 36.5 y, *p* = 0.02), and were more likely to be male (median 88% versus 77% male, *p* < 0.0001) than those excluded. CD4^+^ cell count, plasma viral load, and HIV genotype data for each participant represent the latest pre-therapy measurement collected within 180 d prior to HAART initiation. Ethical approval for this study was granted by the Providence Health Care/University of British Columbia Research Ethics Board.

### Determination of baseline HIV gene sequences and viral subtypes.

HIV RNA was extracted from a single pre-therapy (“baseline”) plasma sample using the QIAGEN (http://www.qiagen.com) viral RNA kit using a BioRobot 9600/9604 or extracted manually using guanidinium-based buffer followed by isopropanol/ethanol washes. The HIV protease (codons 1–99, HXB2 nt 2253–2549), RT (codons 1–400, or for ~25% of sequences codons 1–240 only; nt 2550–3749 or 2550–3269, respectively), Vpr (codons 1–96; nt 5559–5847), and Nef (codons 1–206; nt 8797–9414) were amplified using nested RT-PCR, and “bulk” sequenced in both the 5′ and 3′ directions on an ABI 3700 or 3100 (http://www.appliedbiosystems.com) automated DNA sequencer. HIV sequence data were analyzed using the software Sequencher (Genecodes, http://www.genecodes.com). Nucleotide mixtures were called if the height of the secondary peak exceeded 25% of the height of the dominant peak. Sequence data were aligned to HIV-1 subtype B reference strain HXB2 (Genbank accession number K03455) using a modified NAP algorithm [40]. HIV subtyping was performed by comparing HIV sequence data across HIV protease, RT, and Nef to all known subtype reference sequences in the Los Alamos HIV sequence database (http://hiv-web.lanl.gov/content/hiv-db/mainpage.html). Of total participants, 97.5% harbored subtype B infections. Consensus sequences reflecting the most common amino acid at each codon were generated from pretherapy sequences: these differed from the 2005 HIV-1 subtype B consensus at 1/99 protease, 7/400 RT, 2/96 Vpr, and 7/206 Nef codons, respectively. Genbank accession numbers of all unique HIV sequences used in this study are listed in Text S1.

### Determination of HLA-A, B, and C genotypes by sequence-based typing.

Sequence-based typing (SBT) for HLA-A, B, and C was performed on DNA extracted from a PBMC-enriched frozen blood sample for each participant (*n* = 765). The SBT protocol is a validated “in-house” procedure based on International Histocompatibility Working Group (IHWG) protocols and involves independent, locus-specific, nested PCR amplification of exons 2 and 3 of HLA-A, B, and C followed by automated bidirectional DNA sequencing. Allele interpretation was performed by comparing SBT data against all alleles listed in the IMGT/HLA database (ftp://ftp.ebi.ac.uk/pub/databases/imgt/mhc/hla/) as of August 2005 (Release 2.10). This yields intermediate-to-high level resolution of HLA allele combinations. In order to achieve appropriately sized groups for statistical analysis, HLA alleles were summarized to two-digit resolution; note however that this approach may group together alleles which bind slightly different peptides, thus potentially reducing power to detect HLA-associated polymorphisms in some cases. Ambiguous allele combinations were resolved through incorporation of published allele frequencies and/or haplotype data. HLA-A and B typing was completed for all 765 participants, while HLA-C types were determined for 706 individuals. Although complete ethnicity data are unavailable, class I allele frequencies were consistent with those expected in a predominantly North American white population.

### Identification of HLA-associated amino acid variation across HIV genes.

In order to discriminate between associations likely attributable to viral lineage effects and those that provide evidence for HLA-associated escape or reversion, we adopted the phylogenetically corrected analysis methods described in detail in [16]. Briefly, we used cohort HIV sequences to construct maximum likelihood phylogenetic trees (one for each gene). Since HLA types are available only for the infected individuals sampled, whose sequences form the tips of the tree, we used a maximum likelihood estimate of the sequence at the parental (interior) node proximate to each observation, and counted inferred escape or reversion in these last branches as independent events to be correlated with the HLA of the infected person at the terminal sequence by a Fisher exact test (method 1); alternatively, we used a likelihood ratio test to evaluate whether a model incorporating the effect of HLA association in addition to the phylogenetic structure was significantly better at explaining the data (method 2) [16]. The final list of identified associations represents the union of associations identified by both methods (Table S1).

In order to adjust for multiple comparisons, a *q*-value approach [22], rather than a Bonferroni correction [41], was employed: whereas a Bonferroni correction attempts to limit the probability of even a single false positive (and thus increases the rate of false-negative results), the *q*-statistic sets the proportion of false positives among results identified as significant (the false-discovery rate), an approach which we believe to be more appropriate for gene-wide association scans such as the present one. Associations with *q* < 0.2 (indicating a ~20% false-discovery rate) are presented; in this dataset this corresponded to unadjusted *p*-values 0.0055 > *p* > 3.3x10^−45^ for all genes. Note that the results of the lineage-corrected analysis groups associations into two broad categories based on the direction of the HLA selection pressure. Positive correlations, in which the presence of a specific HLA is associated with the presence of a particular amino acid—or, correspondingly, where the absence of the allele is associated with the absence of the amino acid—are termed “escape” associations, as they presumably reflect the escape variant for that specific HLA allele. Negative correlations, in which the presence of a specific HLA allele is associated with the *absence* of a particular amino acid—or, correspondingly, where a specific amino acid is enriched in the *absence* of a particular HLA allele—are termed “reversion” associations. In this case, the “reversion” amino acid presumably reflects the immunologically susceptible (“wild-type”) form specific for that HLA allele, as well as represents the amino acid most likely to re-emerge following transmission to an individual lacking that HLA allele.

### Mapping identified associations into immune escape maps.

Associations were organized into gene-specific “immune escape maps” whose goal was to capture the minimum number of epitopes (known or putative) required to explain the data. Three sets of escape maps were generated based on the strength of biological evidence supporting each association. The highest level of support was granted to those associations that fell within or proximal to (± 3 aa) a published HLA-restricted epitope (defined as all HLA class 1-restricted ≤ 15-mer epitopes listed in the Los Alamos HIV Immunology database as of December 2006 [23]). HLA-matched associations that fell within these boundaries were grouped together. Note that the ± 3 aa proximal “window” was chosen to identify putative proteasomal processing escape mutations [24–26] based on evidence indicating that the majority of such mutations occur in the three amino acids immediately flanking the epitope [42]. The secondary level of support was granted to associations which fell within or proximal to a known HLA-restricted epitope anchor residue motif (using MotifScan, http://hiv-web.lanl.gov/content/immunology/motif_scan/motif_scan) and/or a putative HLA-restricted epitope identified by an independently validated CTL epitope prediction algorithm (Epipred, http://atom.research.microsoft.com/bio/epipred.aspx [43]) based on scanning the cohort consensus sequence. Again, associations falling within the “motif ± 3 aa flanking window” were grouped together.

If specific amino acid variants were associated with additional HLA alleles in linkage disequilibrium (LD), these alleles were also grouped together within the epitope or motif. To identify HLA alleles in LD, we investigated all possible pairwise allele combinations using a simple Fisher's exact test and conservatively defined all allele pairs with *p* < 0.05 (*q* < 0.2) as linked (Table S2). In cases where LD allele pairs were associated with variation at the same codon, the allele exhibiting the strongest association (as estimated by lowest *p*-value) was classified as the allele driving the association.

To provide in vitro functional support to identified associations, we drew upon a partially published ELISpot dataset of 372 HIV-1 infected, non-white individuals screened for HLA-restricted, CTL-mediated IFN-γ responses against set of 410 overlapping subtype B consensus peptides (OLP) 15 to 20 amino acids in length, spanning the whole expressed HIV-1 subtype B proteome [27]. Associations between possession of individual HLA alleles and responses to specific consensus peptides in the OLP set [27] were assessed by simple Fisher exact test. HLA allele/OLP associations with *p* < 0.05 were considered to be “significantly reactive” and thus indicative that an HLA-restricted CTL epitope lay in the boundaries of that OLP. HLA-associated polymorphisms identified in the present study that mapped directly within an HLA-specific reactive OLP were identified and annotated as “in vitro-supported” on the immune escape maps (green; Figures 2–5). Note that the differences in ethnic composition of the current and ELISpot-characterized [27] study populations may result in an underestimation of in vitro-supported associations, due to differences in cohort HLA composition and thus power to detect significant associations.

## Supporting Information

Figure S1 Illustration of a Case where an Apparent HLA-Associated HIV Amino Acid Variation Pattern Is an Artifact of the Phylogenetic Tree, and Using a Phylogenetic Correction Avoids an Incorrect Assignment of an HLA Driven Association (A) This is a maximum likelihood tree (for details, please see [16]) of the complete HIV Nef sequence set, with the inclusion of several subtyping reference strains from the Los Alamos database. Subtype B sequences are to the right of the node marked “B.” A small number of other HIV subtypes were identified in this cohort, as indicated in the tree. The amino acid at position 18 is tracked through the tree, with the most likely amino acid at each ancestral node estimated. Glutamic acid (E) is the most commonly observed amino acid in this position, and is indicated in red in the tree. However, a large cluster of sequences within subtype B has an Aspartic acid (D) at this position, indicated by gold in the tree (yellow boxed area). There is a paucity of B14 individuals in this subcluster (indicated by magenta lines), giving rise to an apparent “negative” association between E and B14. The apparent statistical significance of this association, if one does not include a correction for the phylogeny, is dominated by this single subcluster, and is the result of lineage effects, not HLA-mediated escape or reversion. This is an example of how sub-lineages within a major subtype can impact association analysis. Based on this type of analysis, as well as statistical estimates of the frequency of immunologically validated associations, we decided to include only phylogenetically corrected associations in this study.
(B) A detail of the yellow boxed area in (A). The probability of a given amino acid being “E” at an interior node is indicated by a number. For example, 9 indicates the probability is greater than 0.9. For the actual sequences at the terminal nodes, this probability is obviously known and if the amino acid is E, the probability is simple 1. However, for the sequences at the interior nodes, the probability is estimated based on the tree topology and evolutionary model. A “0” indicates the probability is less than 0.1 that the amino acid is E, and the color indicates the most likely amino acid at this position any given node in the tree.
(2.2 MB PDF)

Table S1 Full List of HLA Allele-Associated HIV Polymorphisms in Functional and Accessory/Regulatory Proteins Investigated (A) Full list of HLA allele-associated HIV polymorphisms in Nef
(B) Full list of HLA allele-associated HIV polymorphisms in protease, reverse transcriptase, and VPR
“Escape” amino acids indicate amino acids that are enriched in the presence of a specific HLA allele, thus presumably reflecting the escape variant specific for that HLA allele.
“Reversion” amino acids indicate amino acids that are enriched in the *absence* of a specific allele (or likewise, depleted in the presence of a specific HLA allele). “Reversion” amino acids presumably reflect the immunologically susceptible (‘wild-type”) form specific for that HLA allele, and also represent the amino acid to which the sequence may revert upon transmission to an individual lacking that HLA allele.
(518 KB DOC)

Table S2 Full List of HLA Linkage Disequilibrium Pairs Observed in Our Dataset (*p* < 0.05, *q* < 0.2) (30 KB DOC)

Text S1 Accession Numbers (28 KB DOC)

## Abbreviations

aa: amino acid(s)
CTL: cytotoxic T lymphocyte
ELISpot: enzyme-linked immunosorbent spot assay
HLA: human leukocyte antigen
IFN-γ: interferon-gamma
LD: linkage disequilibrium
OLP: overlapping peptide
RT: reverse transcriptase
