# Correction: Evidence of Differential HLA Class I-Mediated Viral Evolution in Functional and Accessory/Regulatory Genes of HIV-1

**DOI:** 10.1371/journal.ppat.0030121

**Published:** 2007-08-31

**Authors:** Zabrina L Brumme, Chanson J Brumme, David Heckerman, Bette T Korber, Marcus Daniels, Jonathan Carlson, Carl Kadie, Tanmoy Bhattacharya, Celia Chui, James Szinger, Theresa Mo, Robert S Hogg, Julio S. G Montaner, Nicole Frahm, Christian Brander, Bruce D Walker, P. Richard Harrigan

doi:10.1371/journal.ppat.0030094

Correction for:

Brumme ZL, Brumme CJ, Heckerman D, Korber BT, Daniels M, et al. (2007) Evidence of differential HLA class I-mediated viral evolution in functional and accessory/regulatory genes of HIV-1. PLoS Pathog 3(7): e94. doi:10.1371/journal.ppat.0030094

Coauthor Tanmoy Bhattacharya's affiliations were listed incorrectly. The correct affiliations are:

**5** Los Alamos National Laboratory, Los Alamos, New Mexico, United States of America,

**6** Santa Fe Institute, Santa Fe, New Mexico, United States of America

(The second affiliation was incorrectly listed as **7** Department of Computer Science and Engineering, University of Washington, Seattle, Washington, United States of America.)

