# Supplementary material for: Evidence of Differential HLA Class I-Mediated Viral Evolution in Functional and Accessory/Regulatory Genes of HIV-1
Source: PLoS Pathog. 2007 Jul 6;3(7):e94. doi: 10.1371/journal.ppat.0030094 (PMC1904471; doi:10.1371/journal.ppat.0030094)

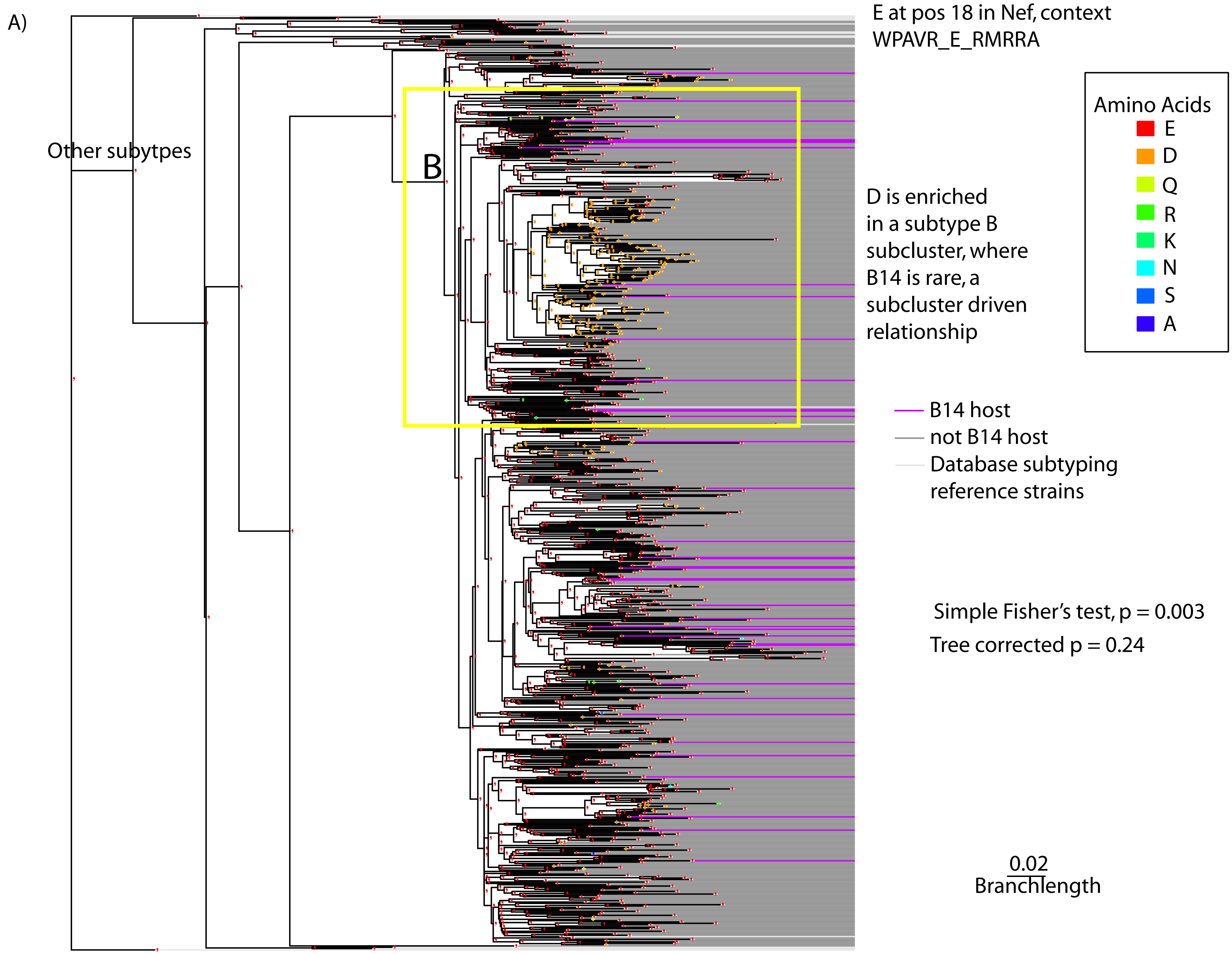
A)
E at pos 18 in Nef, context
WPAVR_E_RMRRA
Other subtypes
B
Amino Acids
E
D
Q
R
K
N
S
A
D is enriched
in a subtype B
subcluster, where
B14 is rare, a
subcluster driven
relationship
B14 host
not B14 host
Database subtyping
reference strains
Simple Fisher's test, p = 0.003
Tree corrected p = 0.24
0.02
Branchlength

B)

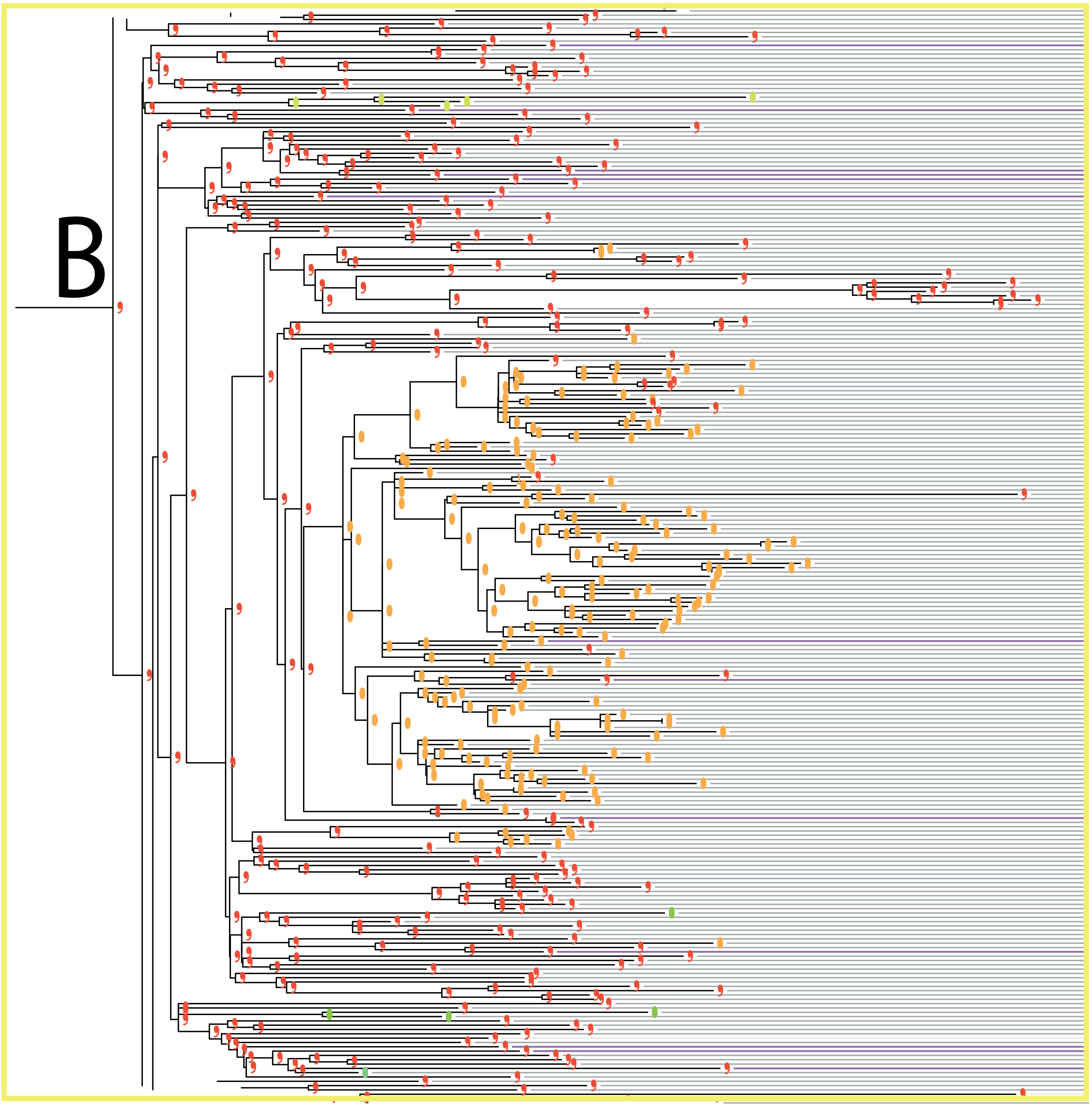

Supplement: Supplementary file 1 [file ppat.0030094.sg001.pdf]
