# Supplementary material for: Evidence of Differential HLA Class I-Mediated Viral Evolution in Functional and Accessory/Regulatory Genes of HIV-1
Source: PLoS Pathog. 2007 Jul 6;3(7):e94. doi: 10.1371/journal.ppat.0030094 (PMC1904471; doi:10.1371/journal.ppat.0030094)
Supplement: Supplementary file 2 [file ppat.0030094.sd001.doc]

Full list of Genbank Accession numbers for HIV sequences featured in this study

PR

EF368373, EF368377, EF368379, EF368380, EF368388, EF368391, EF368393-EF368396, EF368398-EF368402, EF368404, EF368406, EF368408-EF368411, EF368415-EF368421, EF368424-EF368427, EF368429, EF368430, EF368432, EF368438, EF368442-EF368446, EF368448, EF368450, EF368453, EF368454, EF368457-EF368459, EF368461-EF368463, EF368466-EF368474, EF368476, EF368478, EF368479, EF368481, EF368482, EF368485-EF368489, EF368491, EF368493, EF368495, EF368496, EF368499-EF368503, EF368508, EF368510-EF368512, EF368514, EF368516-EF368518, EF368523, EF368524, EF368527, EF368528, EF368530, EF368532-EF368534, EF368536-EF368539, EF368544, EF368545, EF368548, EF368550-EF368555, EF368557, EF368561, EF368565, EF368567, EF368570-EF368575, EF368577, EF368579, EF368580, EF368582, EF368583, EF368585-EF368589, EF368591-EF368597, EF368600, EF368601, EF368603

RT

EF369201, EF369203, EF369204, EF369212, EF369215, EF369217-EF369220, EF369222-EF369226, EF369228, EF369230, EF369232-EF369235, EF369239-EF369245, EF369248-EF369251, EF369253, EF369254, EF369256, EF369262, EF369266-EF369270, EF369272, EF369274, EF369277, EF369278, EF369281-EF369283, EF369285-EF369287, EF369290-EF369298, EF369300, EF369302, EF369303, EF369305, EF369306, EF369309-EF369313, EF369315, EF369317, EF369319, EF369320, EF369323-EF369327, EF369332, EF369334-EF369336, EF369338, EF369340-EF369342, EF369347, EF369348, EF369351, EF369352, EF369354, EF369356-EF369358, EF369360-EF369363, EF369368, EF369369, EF369372, EF369374-EF369379, EF369381, EF369385, EF369389, EF369391, EF369394-EF369399, EF369401, EF369403, EF369404, EF369406, EF369407, EF369409-EF369413, EF369415-EF369421, EF369424, EF369425, EF369427

PRRT

EF368604-EF368611, EF368613-EF368616, EF368621-EF368623, EF368625-EF368629, EF368631-EF368635, EF368637-EF368639, EF368644, EF368646-EF368651, EF368655, EF368656, EF368660, EF368661, EF368664-EF368667, EF368672, EF368673, EF368675-EF368679, EF368681-EF368683, EF368685, EF368687, EF368689, EF368691, EF368694-EF368697, EF368699-EF368706, EF368710, EF368712-EF368717, EF368719-EF368723, EF368725-EF368727, EF368729, EF368734-EF368737, EF368740, EF368741, EF368744, EF368746-EF368748, EF368750-EF368756, EF368758, EF368759, EF368762, EF368763, EF368765-EF368767, EF368769-EF368774, EF368776, EF368779-EF368782, EF368786-EF368795, EF368797, EF368798, EF368800-EF368804, EF368806-EF368812, EF368816, EF368818, EF368819, EF368823, EF368825, EF368826, EF368828, EF368829, EF368831-EF368833, EF368835, EF368839, EF368842, EF368844, EF368848-EF368853, EF368856, EF368858-EF368861, EF368863, EF368869-EF368874, EF368876-EF368878, EF368882, EF368884-EF368892, EF368895, EF368897, EF368900, EF368902-EF368904, EF368906-EF368909, EF368913, EF368918-EF368921, EF368923, EF368924, EF368926, EF368928-EF368930, EF368932, EF368934-EF368936, EF368939, EF368940, EF368942-EF368944, EF368948-EF368950, EF368952-EF368955, EF368957, EF368959, EF368962, EF368964, EF368965, EF368967-EF368969, EF368971-EF368974, EF368976, EF368979, EF368980, EF368982-EF368989, EF368992-EF369000, EF369003-EF369006, EF369010-EF369013, EF369015-EF369022, EF369024, EF369026, EF369028-EF369030, EF369032, EF369033, EF369035, EF369038-EF369044, EF369046, EF369049-EF369052, EF369054-EF369057, EF369059-EF369067, EF369070-EF369072, EF369074-EF369076, EF369081, EF369083, EF369085-EF369088, EF369090, EF369091, EF369093, EF369096, EF369098-EF369105, EF369107-EF369111, EF369113, EF369114, EF369116, EF369118, EF369121, EF369123-EF369127, EF369129, EF369131, EF369134, EF369135, EF369139, EF369140, EF369142-EF369146, EF369149-EF369151, EF369155-EF369161, EF369163-EF369169, EF369171-EF369173, EF369176, EF369177, EF369180, EF369181, EF369183-EF369187, EF369189-EF369192, EF369195-EF369197

NEF

DQ484067-DQ484069, DQ484071-DQ484074, DQ484077-DQ484082, DQ484084, DQ484087, DQ484088, DQ484091-DQ484094, DQ484097, DQ484098, DQ484100-DQ484104, DQ484111, DQ484113, DQ484115, DQ484117, DQ484119, DQ484120, DQ484122-DQ484124, DQ484126, DQ484128, DQ484129, DQ484133-DQ484138, DQ484141-DQ484144, DQ484148, DQ484149, DQ484151, DQ484154, DQ484155, DQ484157, DQ484159, DQ484160, DQ484162-DQ484167, DQ484173, DQ484175, DQ484176, DQ484182, DQ484184-DQ484186, DQ484188-DQ484191, DQ484193-DQ484197, DQ484202-DQ484204, DQ484206-DQ484210, DQ484212-DQ484215, DQ484217, DQ484219, DQ484221, DQ484223, DQ484224, DQ484227, DQ484229, DQ484231-DQ484246, DQ484249, DQ484253, DQ484254, DQ484256-DQ484261, DQ484263, DQ484264, DQ484266-DQ484268, DQ484270, DQ484271, DQ484273-DQ484275, DQ484277, DQ484278, DQ484280, DQ484282, DQ484286-DQ484289, DQ484294-DQ484296, DQ484299-DQ484301, DQ484304, DQ484306, DQ484308, DQ484310, DQ484312-DQ484314, DQ484316-DQ484318, DQ484320, DQ484323-DQ484325, DQ484327, DQ484329, DQ484330, DQ484333-DQ484335, DQ484337-DQ484343, DQ484345-DQ484347, DQ484349, DQ484351, DQ484352, DQ484354, DQ484357, DQ484360-DQ484364, DQ484367, DQ484369-DQ484373, DQ484377-DQ484388, DQ484390-DQ484392, DQ484394-DQ484398, DQ484400-DQ484408, DQ484413, DQ484415, DQ484416, DQ484419, DQ484420, DQ484422-DQ484424, DQ484427, DQ484428, DQ484430, DQ484431, DQ484433, DQ484435-DQ484438, DQ484441, DQ484450, DQ484451, DQ484453, DQ484457-DQ484460, DQ484462-DQ484465, DQ484469-DQ484478, DQ484480, DQ484483, DQ484485-DQ484489, DQ484491, DQ484497-DQ484502, DQ484504, DQ484506, DQ484507, DQ484511, DQ484512, DQ484514, DQ484516-DQ484521, DQ484523-DQ484525, DQ484527, DQ484529, DQ484530, DQ484533, DQ484534, DQ484538, DQ484539, DQ484541-DQ484547, DQ484549-DQ484552, DQ484554-DQ484557, DQ484560, DQ484565, DQ484566, DQ484568, DQ484571, DQ484572, DQ484574-DQ484579, DQ484583, DQ484584, DQ484586-DQ484588, DQ484590, DQ484591, DQ484593-DQ484595, DQ484599-DQ484606, DQ484608-DQ484611, DQ484614-DQ484617, DQ484619, DQ484622, DQ484623, DQ484625-DQ484629, DQ484632, DQ484633, DQ484635, DQ484637-DQ484639, DQ484641, DQ484645-DQ484648, DQ484650, DQ484651, DQ484656-DQ484662, DQ484664-DQ484666, DQ484670, DQ484671, DQ484673, DQ484675-DQ484678, DQ484680-DQ484682, DQ484684-DQ484686, DQ484688-DQ484690, DQ484692, DQ484693, DQ484696, DQ484698, DQ484699, DQ484704-DQ484707, DQ484709, DQ484712, DQ484714-DQ484720, DQ484723, DQ484726-DQ484729, DQ484731-DQ484734, DQ484738, DQ484739, DQ484742-DQ484751, DQ484754-DQ484757, DQ484761-DQ484764, DQ484766-DQ484771, DQ484773-DQ484777, DQ484779-DQ484783, DQ484786, DQ484788-DQ484793, DQ484796, DQ484802-DQ484805, DQ484807, DQ484808, DQ484810, DQ484812-DQ484814, DQ484816-DQ484819, DQ484821-DQ484824, DQ484826, DQ484827, DQ484829, DQ484832-DQ484834, DQ484836-DQ484840, DQ484842, DQ484843, DQ484845, DQ484847-DQ484854, DQ484858-DQ484861, DQ484863-DQ484866, DQ484868-DQ484871, DQ484873, DQ484875-DQ484880, DQ484882-DQ484887, DQ484889, DQ484891, DQ484893-DQ484898, DQ484900, DQ484902, DQ484905, DQ484907, DQ484908, DQ484912, DQ484914-DQ484919, DQ484921, DQ484922, DQ484925, DQ484928, DQ484931, DQ484933, DQ484936-DQ484938, DQ484940, DQ484941, DQ484945, DQ484947-DQ484954, DQ484956-DQ484961, DQ484964-DQ484966, DQ484968, DQ484969, DQ484972, DQ484976-DQ484979, DQ484981-DQ484983, DQ484985, DQ484988, DQ484991, DQ484992, DQ484995, DQ484996, DQ485000-DQ485005, DQ485008, DQ485009, DQ485011, DQ485013, DQ485014, DQ485016, DQ485017, DQ485019, DQ485020, DQ485023-DQ485025, DQ485029, DQ485030, DQ485032-DQ485036, DQ485041-DQ485043, DQ485047-DQ485052, DQ485054-DQ485058, DQ485060, DQ485062-DQ485065, DQ485067-DQ485069, DQ485071-DQ485075, DQ485077, DQ485079-DQ485089, DQ485091, DQ485093, DQ485095-DQ485097, DQ485100, DQ485103-DQ485105, DQ485108, DQ485110-DQ485114, DQ485116-DQ485118, DQ485120, DQ485122, DQ485125-DQ485128

VPR

DQ203856-DQ203858, DQ203861-DQ203865, DQ203867, DQ203869-DQ203871, DQ203873, DQ203875, DQ203879-DQ203881, DQ203883, DQ203886, DQ203888, DQ203891, DQ203893, DQ203895-DQ203898, DQ203902, DQ203903, DQ203907-DQ203909, DQ204309, DQ203910-DQ203912, DQ203915, DQ203916, DQ203919-DQ203928, DQ203930, DQ203933, DQ203934, DQ203936-DQ203940, DQ203942-DQ203946, DQ203948-DQ203950, DQ203954, DQ203957, DQ203959-DQ203962, DQ203964-DQ203966, DQ203968-DQ203971, DQ203973, DQ203975, DQ203976, DQ203978-DQ203980, DQ203982-DQ203985, DQ203987, DQ203989, DQ203990, DQ203993-DQ203995, DQ203998-DQ204008, DQ204010-DQ204019, DQ204022, DQ204026, DQ204027, DQ204030-DQ204035, DQ204042, DQ204045, DQ204046, DQ204051, DQ204052, DQ204055, DQ204056, DQ204058, DQ204062, DQ204064-DQ204067, DQ204069, DQ204074-DQ204076, DQ204079, DQ204080, DQ204084, DQ204086, DQ204088-DQ204090, DQ204092, DQ204093, DQ204095, DQ204097, DQ204098, DQ204100-DQ204103, DQ204105, DQ204107-DQ204109, DQ204112, DQ204116, DQ204117, DQ204119, DQ204122, DQ204124-DQ204127, DQ204129, DQ204131, DQ204133-DQ204135, DQ204138-DQ204140, DQ204144, DQ204146-DQ204153, DQ204156-DQ204158, DQ204160-DQ204162, DQ204164-DQ204167, DQ204169, DQ204170, DQ204172-DQ204174, DQ204178, DQ204179, DQ204181, DQ204183-DQ204188, DQ204190-DQ204195, DQ204197, DQ204198, DQ204201-DQ204204, DQ204206-DQ204211, DQ204213-DQ204216, DQ204218-DQ204221, DQ204223, DQ204225-DQ204229, DQ204231-DQ204235, DQ204237, DQ204238, DQ204240, DQ204243-DQ204248, DQ204251-DQ204253, DQ204256-DQ204260, DQ204262-DQ204265, DQ204267, DQ204269, DQ204271, DQ204272, DQ204274-DQ204278, DQ204280, DQ204281, DQ204283, DQ204284, DQ204289, DQ204291, DQ204292, DQ204295, DQ204297-DQ204300, DQ204302-DQ204305, DQ204307, DQ204308, DQ204310, DQ204311, DQ204314, DQ204318-DQ204322, DQ204327, DQ204328, DQ204330-DQ204333, DQ204335, DQ204339, DQ204340, DQ204342-DQ204347, DQ204353, DQ204354, DQ204358-DQ204367, DQ204370-DQ204374, DQ204376-DQ204380, DQ204383, DQ204384, DQ204387, DQ204388, DQ204391, DQ204393, DQ204395, DQ204396, DQ204398-DQ204400, DQ204402, DQ204405, EF567317-EF567389
