# Supplementary material for: Evidence of Differential HLA Class I-Mediated Viral Evolution in Functional and Accessory/Regulatory Genes of HIV-1
Source: PLoS Pathog. 2007 Jul 6;3(7):e94. doi: 10.1371/journal.ppat.0030094 (PMC1904471; doi:10.1371/journal.ppat.0030094)
Supplement: Supplementary file 3 [file ppat.0030094.st001.doc]

**Supplementary Table 1a: Full list of HLA allele-associated HIV polymorphisms in Nef**

| Gene | Codon | HLA | Escape | Reversion |  | Gene | Codon | HLA | Escape | Reversion |  | Gene | Codon | HLA | Escape | Reversion |  | Gene | Codon | HLA | Escape | Reversion |  | Gene | Codon | HLA | Escape | Reversion |
| --- | --- | --- | --- | --- | --- | --- | --- | --- | --- | --- | --- | --- | --- | --- | --- | --- | --- | --- | --- | --- | --- | --- | --- | --- | --- | --- | --- | --- |
| Nef | 5 | A11 | C |  |  | Nef | 65 | B35 |  | D |  | Nef | 87 | B08 |  | L |  | Nef | 126 | A26 | S |  |  | Nef | 194 | A01 |  | R |
| Nef | 7 | B57 |  | K |  | Nef | 65 | B40 |  | E |  | Nef | 89 | B14 | F | H |  | Nef | 126 | B51 | C | N |  | Nef | 194 | A31 | M | V |
| Nef | 7 | B83 | - |  |  | Nef | 65 | B45 | D | E |  | Nef | 89 | C08 | F | H |  | Nef | 126 | C14 | C | N |  | Nef | 194 | B08 | M | V |
| Nef | 8 | A02 |  | G |  | Nef | 65 | C06 | D | E |  | Nef | 91 | B14 | I | L |  | Nef | 133 | A24 | T | I |  | Nef | 194 | B35 | V | M |
| Nef | 8 | A24 | M |  |  | Nef | 71 | A03 | K | R |  | Nef | 91 | C08 | I | L |  | Nef | 133 | A26 |  | T |  | Nef | 194 | B48 |  | M |
| Nef | 8 | C04 |  | L |  | Nef | 71 | B07 | K | R |  | Nef | 92 | A11 | R | K |  | Nef | 133 | B35 | T | I |  | Nef | 194 | C04 | V |  |
| Nef | 11 | A68 |  | A |  | Nef | 71 | B14 | R | K |  | Nef | 92 | B55 |  | K |  | Nef | 133 | B38 | I | T |  | Nef | 194 | C06 | V |  |
| Nef | 11 | B57 | A | V |  | Nef | 71 | B35 | R | K |  | Nef | 94 | A01 | E, N, Q | K |  | Nef | 133 | B57 | I | T |  | Nef | 196 | A02 | R |  |
| Nef | 11 | C03 |  | K |  | Nef | 71 | C04 | R | K |  | Nef | 94 | B08 | E, M, N, Q | K |  | Nef | 133 | C02 | P |  |  | Nef | 196 | A31 | K | R |
| Nef | 11 | C06 |  | V |  | Nef | 71 | C07 | K | R |  | Nef | 94 | B15 | K |  |  | Nef | 135 | A02 | Y | F |  | Nef | 198 | B15 | L |  |
| Nef | 12 | B40 |  | G |  | Nef | 71 | C08 | R | K |  | Nef | 94 | C03 |  | N |  | Nef | 135 | A03 | Y | F |  | Nef | 198 | B35 |  | Q |
| Nef | 14 | B08 | Y |  |  | Nef | 71 | C16 | R |  |  | Nef | 94 | C07 | E, Q | K |  | Nef | 135 | A11 |  | F |  | Nef | 198 | B57 | M | L |
| Nef | 15 | A31 | D, T | A |  | Nef | 73 | A69 | R | Q |  | Nef | 98 | B40 | D | E |  | Nef | 135 | A24 | F | Y |  | Nef | 198 | C04 |  | Q |
| Nef | 15 | B51 |  | A |  | Nef | 74 | B45 |  | V |  | Nef | 100 | A03 |  | I |  | Nef | 135 | C01 | F | Y |  | Nef | 198 | C14 | K, Q | L |
| Nef | 15 | B57 |  | A |  | Nef | 76 | B81 |  | L |  | Nef | 100 | B40 | M | L |  | Nef | 139 | A24 | L |  |  | Nef | 201 | A33 |  | E |
| Nef | 21 | A11 |  | R |  | Nef | 81 | A29 |  | F |  | Nef | 100 | C03 |  | L |  | Nef | 143 | A23 | Y | F |  | Nef | 201 | B58 |  | E |
| Nef | 21 | A33 | T |  |  | Nef | 81 | A30 |  | F |  | Nef | 101 | B14 | I | V |  | Nef | 143 | B58 |  | F |  | Nef | 202 | B50 |  | Y |
| Nef | 21 | B46 |  | R |  | Nef | 81 | A32 |  | F |  | Nef | 101 | B40 | I | V |  | Nef | 150 | B51 |  | Q |  | Nef | 206 | C02 |  | C |
| Nef | 21 | B58 | T |  |  | Nef | 81 | A33 |  | F |  | Nef | 101 | C01 | V |  |  | Nef | 151 | B39 |  | E |  | Nef | 206 | C03 | * | C |
| Nef | 23 | A34 | Q |  |  | Nef | 81 | A66 |  | F |  | Nef | 101 | C08 | I |  |  | Nef | 151 | B41 | S |  |  | Nef | 206 | C07 | C |  |
| Nef | 23 | B13 | - |  |  | Nef | 81 | B07 |  | F |  | Nef | 102 | A29 | H | Y |  | Nef | 153 | A02 | I | V |  |  |  |  |  |  |
| Nef | 24 | B54 | - |  |  | Nef | 81 | B13 |  | F |  | Nef | 102 | B14 | H |  |  | Nef | 153 | B37 |  | I |  |  |  |  |  |  |
| Nef | 24 | C02 |  | E |  | Nef | 81 | B14 | S |  |  | Nef | 102 | B44 | H | Y |  | Nef | 156 | A30 | D |  |  |  |  |  |  |  |
| Nef | 24 | C06 |  | E |  | Nef | 81 | B18 |  | F |  | Nef | 102 | C08 | H |  |  | Nef | 158 | B40 |  | E |  |  |  |  |  |  |
| Nef | 28 | A11 |  | D |  | Nef | 81 | B35 | F | Y |  | Nef | 102 | C16 | H | Y |  | Nef | 161 | B15 |  | D |  |  |  |  |  |  |
| Nef | 28 | B14 |  | D |  | Nef | 81 | B40 | Y | F |  | Nef | 105 | A01 | R | K |  | Nef | 163 | C16 |  | C |  |  |  |  |  |  |
| Nef | 28 | C05 |  | D |  | Nef | 81 | B41 |  | F |  | Nef | 105 | A02 | K |  |  | Nef | 168 | A31 |  | I |  |  |  |  |  |  |
| Nef | 28 | C08 |  | D |  | Nef | 81 | B42 |  | F |  | Nef | 105 | A03 | Q |  |  | Nef | 168 | C06 | L |  |  |  |  |  |  |  |
| Nef | 30 | A31 |  | V |  | Nef | 81 | B44 |  | F |  | Nef | 105 | B07 | Q |  |  | Nef | 169 | B18 |  | C |  |  |  |  |  |  |
| Nef | 33 | A11 |  | A |  | Nef | 81 | B45 |  | F |  | Nef | 105 | B08 | R | K |  | Nef | 173 | A03 | T |  |  |  |  |  |  |  |
| Nef | 33 | A68 | A | V |  | Nef | 81 | B46 |  | F |  | Nef | 105 | B15 | K |  |  | Nef | 173 | B18 | I | M |  |  |  |  |  |  |
| Nef | 38 | B37 | G | E |  | Nef | 81 | B49 |  | F |  | Nef | 105 | B44 |  | Q |  | Nef | 174 | B40 | D | E |  |  |  |  |  |  |
| Nef | 39 | A24 | K |  |  | Nef | 81 | B52 |  | F |  | Nef | 105 | B49 |  | K |  | Nef | 175 | B44 |  | S |  |  |  |  |  |  |
| Nef | 39 | B37 |  | R |  | Nef | 81 | B55 |  | F |  | Nef | 105 | C07 | Q, R | K |  | Nef | 176 | B44 | T |  |  |  |  |  |  |  |
| Nef | 39 | B44 |  | T |  | Nef | 81 | C04 | F | Y |  | Nef | 105 | C16 |  | Q |  | Nef | 177 | C12 |  | E |  |  |  |  |  |  |
| Nef | 39 | C06 |  | K |  | Nef | 81 | C16 |  | F |  | Nef | 107 | A30 |  | Q |  | Nef | 178 | B40 | R | K |  |  |  |  |  |  |
| Nef | 40 | B37 | R |  |  | Nef | 82 | A03 | R |  |  | Nef | 107 | B13 | R | Q |  | Nef | 182 | A68 |  | I |  |  |  |  |  |  |
| Nef | 43 | B55 | V | I |  | Nef | 82 | B14 |  | K |  | Nef | 107 | C06 | R |  |  | Nef | 182 | A69 | Q |  |  |  |  |  |  |  |
| Nef | 43 | C03 |  | I |  | Nef | 82 | B15 |  | R |  | Nef | 114 | A30 |  | I |  | Nef | 182 | B18 |  | V |  |  |  |  |  |  |
| Nef | 45 | B38 |  | S |  | Nef | 83 | A03 | G | A |  | Nef | 114 | B08 |  | V |  | Nef | 182 | B27 |  | E |  |  |  |  |  |  |
| Nef | 49 | B57 | P |  |  | Nef | 83 | A11 | G | A |  | Nef | 114 | B13 | V | I |  | Nef | 182 | B37 |  | V |  |  |  |  |  |  |
| Nef | 50 | B14 | T |  |  | Nef | 83 | B15 | G | A |  | Nef | 114 | B57 |  | I |  | Nef | 182 | C03 | K |  |  |  |  |  |  |  |
| Nef | 50 | B35 |  | T |  | Nef | 83 | B40 | G | A |  | Nef | 114 | C06 | V | I |  | Nef | 182 | C06 |  | V |  |  |  |  |  |  |
| Nef | 50 | B53 | G | A |  | Nef | 83 | B44 |  | G |  | Nef | 114 | C07 | I | V |  | Nef | 184 | A02 |  | K |  |  |  |  |  |  |
| Nef | 50 | B57 | E |  |  | Nef | 83 | B55 | G | A |  | Nef | 115 | B18 | H | Y |  | Nef | 184 | A36 | E | K |  |  |  |  |  |  |
| Nef | 50 | B58 | D, E | A |  | Nef | 83 | C03 | G | A |  | Nef | 115 | C07 | H | Y |  | Nef | 184 | B14 | K | R |  |  |  |  |  |  |
| Nef | 51 | B58 | N | T |  | Nef | 83 | C07 | A | G |  | Nef | 116 | B57 | N | H |  | Nef | 184 | B27 |  | R |  |  |  |  |  |  |
| Nef | 53 | B14 | P | A |  | Nef | 85 | A02 |  | L |  | Nef | 116 | B58 |  | H |  | Nef | 184 | B57 | R | K |  |  |  |  |  |  |
| Nef | 53 | B81 | S |  |  | Nef | 85 | A11 | L | V |  | Nef | 116 | C06 | N | H |  | Nef | 187 | B39 |  | S |  |  |  |  |  |  |
| Nef | 53 | C08 | P | A |  | Nef | 85 | A68 | L | V |  | Nef | 120 | B51 | F | Y |  | Nef | 188 | A31 |  | R |  |  |  |  |  |  |
| Nef | 54 | B14 | A | D |  | Nef | 85 | B07 | V | L |  | Nef | 120 | C14 | F | Y |  | Nef | 188 | B27 | H | R |  |  |  |  |  |  |
| Nef | 54 | C08 | A | D |  | Nef | 85 | B14 | F, M, R | L |  | Nef | 125 | A30 | H | Q |  | Nef | 188 | C16 | R |  |  |  |  |  |  |  |
| Nef | 56 | B54 | C |  |  | Nef | 85 | B15 | L | V |  | Nef | 125 | B51 |  | Q |  | Nef | 191 | B07 |  | Y |  |  |  |  |  |  |
| Nef | 58 | A33 | V | L |  | Nef | 85 | B55 | L | V |  | Nef | 125 | C07 |  | H |  | Nef | 191 | B14 |  | F |  |  |  |  |  |  |
| Nef | 61 | C05 |  | Y |  | Nef | 85 | C03 | L | V |  | Nef | 125 | C14 |  | Q |  | Nef | 191 | C08 |  | F |  |  |  |  |  |  |
| Nef | 62 | A74 | S |  |  | Nef | 85 | C07 | V | L |  |  |  |  |  |  |  | Nef | 192 | B39 | R | H |  |  |  |  |  |  |
|  |  |  |  |  |  | Nef | 85 | C08 | F, M, R | L, V |  |  |  |  |  |  |  |  |  |  |  |  |  |  |  |  |  |  |

**Supplementary Table 1b: Full list of HLA allele-associated HIV polymorphisms in Protease, Reverse Transcriptase and VPR**

| Gene | Codon | HLA | Escape | Reversion |  | Gene | Codon | HLA | Escape | Reversion |  | Gene | Codon | HLA | Escape | Reversion |
| --- | --- | --- | --- | --- | --- | --- | --- | --- | --- | --- | --- | --- | --- | --- | --- | --- |
| PR | 10 | B15 | I | L |  | RT | 11 | B40 | R | K |  | VPR | 28 | B40 | H |  |
| PR | 12 | B51 |  | T |  | RT | 11 | C03 | R | K |  | VPR | 32 | B27 | K | R |
| PR | 12 | B52 | A | T |  | RT | 35 | B57 |  | I |  | VPR | 32 | C01 | K | R |
| PR | 14 | A68 | R | K |  | RT | 102 | B48 | R | K |  | VPR | 32 | C02 | K | R |
| PR | 14 | B51 | R | K |  | RT | 102 | C08 | R |  |  | VPR | 37 | B51 | A | V |
| PR | 14 | C14 | R | K |  | RT | 123 | B35 | E | D |  | VPR | 37 | C14 | T |  |
| PR | 14 | C15 | R | K |  | RT | 123 | B44 | D | E |  | VPR | 48 | A25 |  | E |
| PR | 15 | B51 | V | I |  | RT | 123 | C04 |  | D |  | VPR | 55 | A33 | T | A |
| PR | 35 | B44 | D | E |  | RT | 135 | A02 |  | V |  | VPR | 63 | A02 | T |  |
| PR | 35 | C05 | D | E |  | RT | 135 | A25 |  | T |  | VPR | 63 | A26 |  | I |
| PR | 35 | C16 |  | E |  | RT | 135 | A29 |  | T |  | VPR | 63 | B38 | V |  |
| PR | 37 | C16 | S |  |  | RT | 135 | B13 |  | T |  | VPR | 84 | A29 |  | T |
| PR | 63 | B13 | S | P, C |  | RT | 135 | B51 | T | I |  | VPR | 84 | B50 |  | I |
| PR | 64 | B13 | M |  |  | RT | 135 | B58 |  | T |  | VPR | 84 | C16 |  | T |
| PR | 93 | B15 | L | I |  | RT | 135 | C08 |  | T |  | VPR | 85 | A31 |  | Q |
|  |  |  |  |  |  | RT | 135 | C14 | T | I |  | VPR | 86 | C02 |  | Q |
|  |  |  |  |  |  | RT | 135 | C15 | T | I |  | VPR | 86 | C06 | P |  |
|  |  |  |  |  |  | RT | 162 | B07 | C | S |  | VPR | 87 | C01 |  | R |
|  |  |  |  |  |  | RT | 165 | B07 | I | T |  | VPR | 88 | C01 | G |  |
|  |  |  |  |  |  | RT | 173 | C07 |  | T |  | VPR | 93 | A25 | S |  |
|  |  |  |  |  |  | RT | 174 | B15 | R | Q |  |  |  |  |  |  |
|  |  |  |  |  |  | RT | 177 | B35 | E | D |  |  |  |  |  |  |
|  |  |  |  |  |  | RT | 200 | B08 | T |  |  |  |  |  |  |  |
|  |  |  |  |  |  | RT | 200 | B40 | I |  |  |  |  |  |  |  |
|  |  |  |  |  |  | RT | 203 | A29 | D | E |  |  |  |  |  |  |
|  |  |  |  |  |  | RT | 207 | B15 | E, R | Q |  |  |  |  |  |  |
|  |  |  |  |  |  | RT | 211 | B44 |  | R |  |  |  |  |  |  |
|  |  |  |  |  |  | RT | 245 | B57 | E | V |  |  |  |  |  |  |
|  |  |  |  |  |  | RT | 245 | B58 | E | V |  |  |  |  |  |  |
|  |  |  |  |  |  | RT | 245 | C06 |  | V |  |  |  |  |  |  |
|  |  |  |  |  |  | RT | 250 | B53 |  | D |  |  |  |  |  |  |
|  |  |  |  |  |  | RT | 275 | C17 |  | K |  |  |  |  |  |  |
|  |  |  |  |  |  | RT | 277 | A02 | K | R |  |  |  |  |  |  |
|  |  |  |  |  |  | RT | 277 | A03 | R | K |  |  |  |  |  |  |
|  |  |  |  |  |  | RT | 309 | A31 |  | I |  |  |  |  |  |  |
|  |  |  |  |  |  | RT | 321 | A66 |  | P |  |  |  |  |  |  |
|  |  |  |  |  |  | RT | 321 | B81 |  | P |  |  |  |  |  |  |
|  |  |  |  |  |  | RT | 329 | C05 |  | I |  |  |  |  |  |  |
|  |  |  |  |  |  | RT | 335 | C02 | D |  |  |  |  |  |  |  |
|  |  |  |  |  |  | RT | 345 | A11 |  | P |  |  |  |  |  |  |
|  |  |  |  |  |  | RT | 369 | A30 |  | T |  |  |  |  |  |  |
|  |  |  |  |  |  | RT | 369 | B13 |  | T |  |  |  |  |  |  |
|  |  |  |  |  |  | RT | 376 | C12 | S |  |  |  |  |  |  |  |
|  |  |  |  |  |  | RT | 379 | B38 | G |  |  |  |  |  |  |  |
|  |  |  |  |  |  | RT | 379 | B58 | G | S |  |  |  |  |  |  |
|  |  |  |  |  |  | RT | 386 | B53 | I | T |  |  |  |  |  |  |
|  |  |  |  |  |  | RT | 399 | A32 | D | E |  |  |  |  |  |  |
