# Supplementary material for: Evidence of Differential HLA Class I-Mediated Viral Evolution in Functional and Accessory/Regulatory Genes of HIV-1
Source: PLoS Pathog. 2007 Jul 6;3(7):e94. doi: 10.1371/journal.ppat.0030094 (PMC1904471; doi:10.1371/journal.ppat.0030094)
Supplement: Supplementary file 4 [file ppat.0030094.st002.doc]

**Supplementary Table 2:**

Full list of HLA linkage disequilibrium pairs observed in our dataset (p<0.05, q<0.2)
